# To Correspondents

**Published:** 1873-10

**Authors:** 


### Editorial.

***To Correspondents.***

Induced by an earnest desire to comply with the wishes and advance the interests of correspondents, we have occasionally admitted to the pages of the JOURNAL communications which our judgment, unbiased, would have induced us to reject. To protect our pages from the introduction of any matter not in the highest degree worthy, and to secure to our readers that to which they are justly entitled, *i. e.*, original matter from reliable sources, we shall hereafter publish no article from an unknown source unless it be accompanied by a reference to some resident practitioner in good standing who will vouch for the good faith and respectability of the writer.

The JOURNAL will not be degraded into an advertising medium for individual pretension, but a permanent record of professional progress.

